# Nurses’ experiences of a screening and associated psychosomatic consultation service for mental comorbidities in somatic care inpatients – a qualitative study

**DOI:** 10.3389/fpsyt.2023.1148142

**Published:** 2023-06-02

**Authors:** Lea-Elena Braunschneider, Johannes Seiderer, Siobhan Loeper, Bernd Löwe, Sebastian Kohlmann

**Affiliations:** Department of Psychosomatic Medicine and Psychotherapy, University Medical Center Hamburg-Eppendorf, Hamburg, Germany

**Keywords:** anxiety, depression, nurse, Patient Health Questionnaire-4, screening, psychosomatic consultation/liaison service, qualitative study

## Abstract

**Background:**

Screening for mental comorbidities and related liaison service can reduce hospital length of stay in somatic hospital care. To develop, test and sustain such health care services, stakeholder feedback is required. One of the most important stakeholders in general hospital care and health care processes are nurses.

**Aim:**

The aim of this study is to explore nurses’ experiencess on standardized nurse-led screening for mental comorbidities and associated psychosomatic consultation service in routine somatic inpatient care.

**Method:**

Semi-structured qualitative interviews were conducted with 18 nurses that were involved in a nurse-led screening service for mental comorbidities on internal medicine or dermatological wards. Data were analyzed using thematic analysis.

**Results:**

Eight thematic groups were developed. On the one hand, participants reported benefits of screening: mental health education, general mental health awareness, holistic treatment approach, opportunity to build rapport with patients and reduction in workload. On the other hand, possible psychological effects of the intervention, reasons why patients may not want to be referred and application requirements to facilitate delivery were identified. None of the nurses opposed screening and associated psychosomatic consultation service.

**Conclusion:**

All nurses endorsed the screening intervention and considered it meaningful. Nurses particularly emphasized the potential for holistic patient care and nurses’ improved skills and competencies, but partly critizised current application requirements.

**Relevance to clinical practice:**

This study adds on existent evidence on nurse-led screening for mental comorbidities and associated psychosomatic consultation service by emphasizing its potential to improve both patient care as well as nurses’ perceived self-efficacy and job satisfaction. To take full advantage of this potential, however, usability improvements, regular supervision, and ongoing training for nurses need to be considered.

## Background

Approximately one out of six inpatients in somatic hospital care suffers from comorbid mental disorders like anxiety or depression (1). Studies indicate that half of these cases are not properly recognized (2) and consequently not treated in line with guidelines (3). In addition, unrecognized mental comorbidities in general somatic hospital care predict worse outcomes for somatic disorders and prolong overall hospital stays (4). International guidelines emphasize the importance of early detection for depression in patients, including those with somatic illnesses and in high-risk populations (5–7). Recently, a draft recommendation from the U.S. Preventive Service Task Force (8) on screening for anxiety was published. Notably, studies suggest that screening for mental comorbidities should be embedded where an adequate referral system is in place (9). According to recent systematic reviews, it appears that mental health screening and related liaison service can reduce hospital length of stay (10, 11). Such mental health screenings and related liaison services, are a complex intervention, and therefore, require process evaluation to develop, test, and sustain (12). To maximize the potential of such promising interventions, guidelines recommend stakeholder feedback (13). Although stakeholder feedback is becoming more common in health care and is widely recognized as an important element, unsatisfactuary attention is paid to it (14).

One of the most important stakeholders in general hospital care and health care processes are nurses (15), as they are the first and closest contact to inpatients (16). This position allows them to provide a wide range of services (17). However, their attitude not only influences the success of general medical services (18), but their perceptions and willingness is critical to the successful delivery of screening (19). Despite having a gatekeeper position, nurses´ perceptions on screening and associated mental health services have not been investigated yet. Therefore, the purpose of this study is to determine nurses’ experiencess of a screening and associated psychosomatic consultation service in routine somatic inpatient care.

## Methods

### Context of the study

This qualitative study was conducted at the University Medical Center Hamburg-Eppendorf (UKE), Germany, within the framework of a standardized nurse-led screening for mental comorbidities and an associated psychosomatic consultation service implemented on internal medicine and dermatological wards in 2018. The initiation of this service was requested by the departments of internal medicine and dermatology. In Germany, mental health services in internal medicine are closely linked to psychosomatic medicine. Internationally, this service is often referred to as psychiatric consultation or liaison service. The special focus of this service is the bio-psycho-social perspective with a psychotherapeutic approach. The Patient Health Questionaire-4 (PHQ-4) is used as screening tool. It is a very well evaluated, ultra-short questionnaire consisting of two core items to identify individuals who may be suffering from depression and/or anxiety disorder (20). As one core component of the standardized nurse-led screening for mental disorders the PHQ-4 is administered at each inpatient admission interview. Nurses are instructed to conduct the screening at each inpatient admission interview by reading the questions aloud and ticking the answers. If the PHQ-4 indicates an elevated screening score (PHQ-4 score ≥ 4 points), the affected patient is asked whether he/she would like help from a psychosomatic consultation service. The procedure is supposed to take less than 2 minutes.Through an electronic referral system, the Department of Psychosomatic Medicine is notified, and a mental health specialist (MD for psychosomatic medicine or registered clinical psychologist) conducts a same-day psychosomatic consultation. The consultation depends on the exacteration of each case and ranges from 30 to 60 min and is paid in-house. If the positive screening result is validated within the clinical consultation, further treatment can be initiated at the University Medical Centre Hamburg-Eppendorf. Basically, this psychosomatic approach refers to the interplay of mental and physical health.

Prior to implementation, psychotherapists from the Department of Psychosomatic Medicine and Psychotherapy trained nurses in the screening process. Nurses were trained at least twice for about one hour on each ward. The standardized implementation of the screening, but also the handling of potentially difficult reactions of the patients (e.g., patient cries or gets angry) were practiced and possible stigmatization expectations were discussed. After implementation, the staff of the Department of Psychosomatic Medicine and Psychotherapy were available for further inquiries. Kohlmann et al. describe the screening and associated psychosomatic consultation services in more detail (21).

### Study development, interview guideline and questionnaires

The design and methods applied the consolidated criteria for qualitative research (COREQ) (22). We developed a semi-structured interview guide with open and broad questions to capture aspects of knowledge and experience with all components of the screening and associated psychosomatic consultation service and to get access to general opinions and attitudes regarding the holistic care of patients with a primary somatic reason for treatment. Following informed consent, self-reported demographic information (gender, age, education, employment status, sick days in the past month), seven additional questions about personal experiences with mental health and usage of the PHQ-4 were collected from each participant.

### Recruitment and interview procedure

We invited all nurses working on the cooperating internal medical and dermatological wards of the University Medical Center Hamburg-Eppendorf to participate through promoting the project during team meetings, flyers in commen rooms and direct inquiries. As such, not all nurses could be approached personally. All nurses who conducted the screening could participate. Prior to the interviews, all participants gave written informed consent and agreed that single phrases of the interview might be used for publication. Participants were told that they could stop the interview at any time if they felt uncomfortable about the questions. Nurses received no reimbursement for participation. LEB (MSc. Psychology) or JS (MD candidate) conducted the interviews individually and face-to-face. Participants did not know the interviewers prior to the interview. Interviews were conducted in accordance with the interview guideline from June 2021 to August 2021.

## Data analysis

Following the rules of Dresing and Pehl (23), experienced student research assistants (B.Sc. Psychology) transcribed the interviews verbatim, and LEB checked the transcripts for accuracy. Qualitative analysis was supported by the MAXQDA software package (version 2020).We use thematic analysis by Braun and Clarke (24), within the framework of an essentialist approach, meaning that we considered our participants’ words as direct access to their experiences. Led by the research question, we used an inductive approach and coded data semantically.

Following Braun and Clark’s rules, LEB (female, MSc. Psychology, PhD candidate and experienced with qualitative research) familiarized herself with the data and began coding parts of the entire dataset. These codes were organized into themes, and reviewed repeatedly with respect to the entire dataset. Through several discussions with SK (male clinical psychologist, psychotherapist and senior researcher with experience in qualitative research) and JS (male medical student, MD candidate), we reached consensus regarding citations, codes, and themes. We were aware, that the perspective and beliefs of the researcher could shape the research process and acknowledged our active part during data analysis. Nevertheless, we tried to work to the best of our knowledge and belief and have followed the checklist of criteria for good thematic analysis (24).

## Results

### Participants

A total of 19 nurses agreed to participate in the study. One nurse refused to be included after giving written consent due to doubts about the anonymization process. Of 18 nurses interviewed, 14 were female; mean age of all were 34.9 (SD = 11.5) years on average. Age ranged from 23 to 59 years. Seven nurses worked in the dermatology ward and 12 worked in the internal medicine ward. Table 1 shows the age group and additional data on personal experience with mental health and use of the PHQ-4 for each participant. On average, nurses perform 7.6 screenings per week, ranging from 1 to 18 times. Some participants reported personal experiences with mental health problems. Half of the nurses reported being trained to work with people suffering from mental health problems. Although half of the nurses reported having been trained to use the PHQ-4, 14 of them felt adequately trained. Fifteen nurses indicated that they had adequate time in their work routine to use the PHQ-4. Every nurse felt the PHQ-4 was useful and would continue to use it.

**Table 1:** Participants’ age group, personal experience with mental health and PHQ-4 use.

Nurse ID	Age group, years	Average use of the PHQ-4 per week	Have you or a family member ever been or are currently affected by mental health problems?	Have you been trained to deal with people with mental health problems?	Have you been trained to use the PHQ-4?	Do you feel adequately trained to use the PHQ-4?	Do you feel that you have enough time in your work routine to use the PHQ-4?	Do you feel that using the PHQ-4 is meaningful?	Would you like to continue using the PHQ-4?
1	30–39	5		✓	✓	✓	✓	✓	✓
2	50–59	5		✓	✓	✓	✓	✓	✓
3	30–39	3	✓	✓	✓	✓	✓	✓	✓
4	30–39	13		✓	✓	✓	✓	✓	✓
5	20–29	10	✓	✓	✓	✓	✓	✓	✓
6	20–29	8	✓	✓	✓	✓	✓	✓	✓
7	30–39	6		✓	✓	✓	✓	✓	✓
8	20–29	4	✓	✓	✓	✓	✓	✓	✓
9	20–29	13		✓	✓	✓	✓	✓	✓
10	20–29	10	✓	✓	✓	✓	✓	✓	✓
11	20–29	1	✓	✓	✓	✓	✓	✓	✓
12	50–59	2		✓	✓	✓	✓	✓	✓
13	30–39	18	✓	✓	✓	✓	✓	✓	✓
14	30–39	5		✓	✓	✓	✓	✓	✓
15	50–59	2		✓	✓	✓	✓	✓	✓
16	40–49	6		✓	✓	✓	✓	✓	✓
17	20–29	15		✓	✓	✓	✓	✓	✓
18	20–29	12		✓	✓	✓	✓	✓	✓

### Identified themes

We identified eight overarching themes. An overview of all themes and codes with citation examples is provided in Table 2.

**Table 2:** Themes, codegroups and example quotes.

Theme	Codegroup	Example quote
Education in mental health	Understanding psychosomatic approach	“I did not initially understand why we were doing [this]. But the more I [...] deal with these patients, the more I understand why this [screening] is important and how psychological burden develops.” (Nurse #10)
	Openess to mental health	“[Mental burden] used to be a taboo subject. [...] However, now I can deal with it better [...]. I am far more open now.” (Nurse #13)
Mental health awareness	General awareness	“On other wards I have seen that mental burden is often neglected and just not taken into account [...]. I think it is good to give some attention to mental health.” (Nurse #8)
	Patients’ self-awareness	“[Patients] might think about whether they are [psychologically] burdened? I think some [...] do not even reflect on how they are doing at the moment.” (Nurse #9)
	Emotional relief	“[Patients] just get something off their chest.” (Nurse #5)
Holistic treatment	Identifying undetected burdens	“There are hidden problems [that] have nothing to do with the disease [for which the patient is hospitalized]. Which I as a nurse do not know about [...], so this should be done on every ward.” (Nurse #13)
	Body and mind belong together	“It belongs together. Body and mind are one.” (Nurse #2) “If the psyche is healthy, it is easier to become physically healthier, and vice versa.” (Nurse #8)
	Normalizing mental illness	“It is important to make patients understand that it is not something rare or that they are not alone, that many people face something like this at some point in their life.” (Nurse #11)
	Changes in help-seeking behavior in patients	“Right after the screening, one patient said, ‘Yes, I think I need help.’ I think she realized at that very moment that she was not well.” (Nurse #13)
Building up rapport	Point of contact	“[We nurses] are the right point of contact. [...] I feel like we just have more contact with [...] patients and they open up to us.” (Nurse #9)
	Icebreaker to talk about mental health	“[Screening questions are an] icebreaker to get into dialog.” (Nurse #18)	Building up a relationship	“In nursing, the somatic or the physical is not as intimate anymore. However, the person themselves, what they are thinking and what their feelings are, that is a whole other level. And when you talk about these feelings, this has a lot to do with building up rapport.” (Nurse #8)
Work relief	Low threshold access to help	“[Patients] get the opportunity or an offer and that’s a lot easier for some already.” (Nurse #16)
	Scheduling a consultation as act of care	“When scheduling a [psychosomatic] consultation [...] it helps patients and us: patients feel [better] because of the consultation and we can take better care of them or have already taken better care of them.” (Nurse #17)
	Professional support	“Then someone [a mental-health professional] comes, the [colleague] just comes for this particular patient and takes his time [for the patient].” (Nurse #1)
	Enhancing self-efficacy through nurse involvement	“That even we as nurses can order a consultation [...] That my clinical judgment is taken into account - I think that is great “(Nurse #13)
	Effortless due to time efficiency	“The time factor is usually so low that [the screening] is not a load.” (Nurse #10)
Psychological effects	Questions can cause discomfort	“I think such questions are generally more difficult to answer than others [...]. But that does not stop me from asking them.” (Nurse #11)
	Patients´ fear of labeling	“As soon as it goes in any psychological direction, [patients] immediately close down. […] Only crazy people go there [psychiatry].” (Nurse #16)
	Questions can trigger emotional response	“[One patient] immediately had tears running down his face when I started asking him this questionnaire. I felt weighed [because] I felt sorry for him.” (Nurse #7)
	Gratitude and appreciation toward the health offer	“There are also many [patients] [...] who do not dare to say, “I need help.” And when I then ask them, sometimes they even beam.” (Nurse #13)
Reasons not wanting referral	Get help elsewhere	“[Patients] have other resources that they would be more likely to use. Like friends and family.” (Nurse #13)
	Patients need time to process	“[The screening result is] too much in the context of the admission interview. And [the patient] first needs a day to clarify this with himself.” (Nurse #14)
	Lack of information about the offered help	“[Patients] do not know what the psychosomatic counseling is.” (Nurse #9)	
	Psychological burden is trivialized	“[Patients] tend to sweep it [their psychological burden] under the carpet.” (Nurse #9).
	One should not talk about it	“[People] have been brought up not to talk about [psychological burden]. It remains behind closed doors.” (Nurse #1)
	Mental issues are not tangible	“This has to do with fear [...] and is tainted with shame. People do not talk about it. I have a problem with my liver and kidneys. It is something that is detectable.” (Nurse #1)
	Inhibition threshold is too high	“Patients often refuse help because it seems that the barrier to accept help [for psychological burden] is higher than for other [somatic] illnesses or problems.” (Nurse #11)
Application requirements	Private setting and one-on-one conversation	“If there is another patient in the room [...] then I always have the feeling that the patient cannot answer honestly and I always have such a strange feeling. I do not like having those conversations then as much as when I am alone with the patient.” (Nurse #17)
	Obstacles in patient communication	“If there is a slight language barrier, or slight cognitive limitations, then [conducting the screening] is not so easy.” (Nurse #11)
	Patient-centered language	“[Rephrasing PHQ-4 questions] makes it sound a little nicer.” (Nurse #9)
	Voluntary participation	“[The whole process] remains a voluntary thing for the patient. [...] The patient has the power to choose until the end.” (Nurse #1)

### Education in mental health

Many nurses reported that they experienced educational gains related to mental health as a result of conducting the screening. As one of them described: “I did not initially understand why we were doing [this]. But the more I [...] deal with these patients, the more I understand why [screening] is important and how psychological burden develops” (nurse #10). Furthermore, nurses recounted that they had become more open in dealing with mental health problems. Nurse #13 recapitulated “[mental burden] used to be a taboo subject. [...] However, now I can deal with it better [...]. I am far more open now” (nurse #13).

### Mental health awareness

One nurse shared that the screening creates a general awareness of mental health within care: “On other wards I have seen that mental burden is often neglected and just not taken into account [...]. I think it is good to give some attention to mental health” (nurse #8). Moreover, nurses noted that through the screening patients “might think about whether they are [psychologically] burdened? I think some [...] do not even reflect on how they are doing at the moment” (nurse #9). Thus, patients are given an impetus to reflect on their inner selves and can experience emotional relief and “just get something off their chest” (nurse #5) in the context of the psychosomatic consultation.

### Holistic treatment

Many nurses recognized that screening for mental health comorbidities in primarily somatic inpatients contributes to a holistic approach to treatment, for example, by uncovering undetected psychological burden. One nurse summarized: “There are hidden problems [that] have nothing to do with the disease [for which the patient is hospitalized]. Which I as a nurse do not know about [...], so this should be done on every ward” (nurse #13). One nurse declared: “It belongs together. Body and mind are one” (nurse #2). For example, nurses in the dermatology ward highlighted the relationship between skin and psyche: “Symptoms like itching become worse in emotional situations, under stress. [...] You can see that there is a connection.” (nurse #10). Nurse #8 concluded: “If the psyche is healthy, it is easier to become physically healthier, and vice versa.”

Another nurse stated that the screening could help to normalize mental illness: “It is important to make patients understand that it is not something rare or that they are not alone, that many people face something like this at some point in their life” (nurse #11). One nurse recalled a specific case: “Right after the screening, one patient said, ‘Yes, I think I need help.’ I think she realized at that very moment that she was not well” (nurse #13). This eventually led the patient to seek professional help.

### Building up rapport

Nurses reported that they “are the right point of contact. [...] I feel like we just have more contact with [...] patients and they open up to us” (nurse #9). They feel the screening is an “icebreaker to get into dialog” (nurse #18) and to talk about mental health. In this context, one nurse told us: “In nursing, the somatic or the physical is not as intimate anymore. However, the person themselves, what they are thinking and what their feelings are, that is a whole other level. And when you talk about these feelings, this has a lot to do with building up rapport” (nurse #8).

### Work relief

Nurses named several aspects that make their nursing work easier and less stressful. Being able to offer help for mental health issues was reported as beneficial not only for patients, who “get the opportunity or an offer and that’s a lot easier for some already,” (nurse #16). But for nurses, too: “when scheduling a [psychosomatic] consultation [...] it helps patients and us: patients feel [better] because of the consultation and we can take better care of them or have already taken better care of them” (nurse #17). The possibility to get professional support was perceived as facilitating: “Then someone [a mental-health professional] comes,” takes over the psychosomatic consultation service and “the [colleague] just comes for this particular patient and takes his time [for the patient].” (nurse #1).

Beyond that, screening appears to strengthen nurses’ self-efficacy: “That even we as nurses can order a consultation [...] That my clinical judgment is taken into account - I think that is great” (nurse #13). In addition, nurse #10 reported, “the time factor is usually so low that [the screening] is not a load.”

### Psychological effects

Nurses told us that questions about psychological burden would sometimes result in psychological effects. One nurse pointed out: “I think such questions are generally more difficult to answer than others [...]. But that does not stop me from asking them” (nurse #11). Nurses reported discomfort when there were commonalities with patients (e.g., same age): “Especially in the beginning I was apprehensive [...] about asking patients who were my age” (nurse #6).

Concurrently, there were reports of suspected negative effects for patients. Some nurses worried that patients may feel labeled when being asked: “As soon as it goes in any psychological direction, [patients] immediately close down. […] Only crazy people go there [psychiatry]” (nurse #16). Sometimes questions about psychological burden may trigger emotional reactions. One nurse shared an experience with an elderly man who “immediately had tears running down his face when I started asking him this questionnaire.” The nurse described that she “felt weighed [because] I felt sorry for him” (nurse #7). Nevertheless, most nurses reported that they perceived gratitude and appreciation of the patients toward the health offer, because “there are also many [...] who do not dare to say, “I need help.” And when I then ask them, sometimes they even beam” (nurse #13).

### Reasons not wanting referral

Some nurses speculated about why some patients may not want psychosomatic counseling. For example, nurses explained that patients “have other resources that they would be more likely to use. Like friends and family” (nurse #13). Alternatively, patients need more time after the screening result, because that is “too much in the context of the admission interview. And [the patient] first needs a day to clarify this with himself” (nurse #14). Or, there is a lack of information and the patients “do not know what the psychosomatic counseling is” (nurse #9) which leads them to refuse treatment in general. Other nurses observed that patients tend to trivialized their own mental health problems and “tend to sweep it [their psychological burden] under the carpet” (nurse #9).

One transgenerational reason could be that “[people] were brought up not to talk about [psychological burden]. It remains behind closed doors” (nurse #1). Nurse #1 further explained that mental health problems are not tangible and that “this has to do with fear [...] and is tainted with shame. People do not talk about it. I have a problem with my liver and kidneys. It is something that is detectable.” This is accompanied by the “experience that patients often refuse help because it seems that the barrier to accept help [for psychological burden] is higher than for other [somatic] illnesses or problems” (nurse #11).

### Application requirements

Some nurses pointed out common pitfalls when performing the screening and suggested ways to ensure smooth enforcement. As questions about psychological burden can be perceived as challenging, nurses felt that screening should be conducted in a private setting within a one-on-one conversation: “If there is another patient in the room [...] then I always have the feeling that the patient cannot answer honestly and I always have such a strange feeling. I do not like having those conversations then as much as when I am alone with the patient” (nurse #17). Moreover, the screening questions themselves can be a stumbling block. Nurses reported that “if there is a slight language barrier, or slight cognitive limitations, then [conducting the screening] is not so easy” (nurse #11). Therefore, some proposed more patient-centered language. For example, screening terminology could be simplified with “simple “yes” or “no” answers” (nurse #12) instead of the four-point scale. Other nurses reported that they already rephrased PHQ-4 questions to create a more personal approach, “that makes it sound a little nicer” (nurse #9), or to clarify the intervention, “I ask if there is a “need to talk,” because I think a lot of people assume that “help for these problems” is a proper therapy” (nurse #8). Finally, a reference was made to the voluntary nature of the offer of assistance. One nurse suggested, “you may ask patients in advance if they would like to be asked such psychological questions” (nurse #3), with another succinctly stating, “[the whole process] remains a voluntary thing for the patient. [...] The patient has the power to choose until the end” (nurse #1).

## Discussion

In this qualitative study, we sought to understand nurses’ experiencess on an existing standardized nurse-led psychosomatic screening and associated psychosomatic consultation service for mental health comorbidities in somatic care inpatients. Overall, none of the nurses interviewed opposed screening and associated psychosomatic counseling for mental comorbidities in somatic care inpatients. The results can be qualitatively grouped into eight thematic groups; on the one hand, the nurses interviewed were able to cite specific benefits, such as mental health education, general mental health awareness, holistic treatment approach, opportunity to build rapport with patients, and reduction in workload, but on the other hand, they also pointed out that the intervention may have psychological effects, cited reasons why patients may not want to be referred, and indicated that application requirements should be modified to facilitate delivery.

Already 20 years ago, the World Health Organization (25) emphasized the importance of well-trained nurses who have knowledge, competence, and confidence in mental health. Consistent with other studies, participating nurses reported that conducting the screening and working with affected patients was educational for them to understand the psychosomatic approach (26). Indeed, such experiences concur with McInnes, Halcomb (27) findings that personal experience with people suffering from mental health problems contributes to a deeper understanding of mental disorders than actual formal training, aiding in a more open-minded approach to mental health. Furthermore, the interviewed nurses reported that standardized screening improves mental health awareness in terms of general awareness in care, thus benefiting patients. The nurses reported that improved awareness allows them to address patients’ fears and concerns, which according to Gausvik et al. (28), increases nurses’ effectiveness, improves job satisfaction, and ultimately improves patient outcomes.

According to our results, standardized nurse-led screening for mental health comorbidities on somatic wards contributes to a holistic treatment approach. Indeed, studies of similar interventions, e.g., in hospitalized cardiac patients or in perinatal care, have resulted in significant clinical improvement in patient outcomes (29–34). As our results already show, undetected cases are detected and according to Halcomb et al. (35), the ability to identify mental health problems early and offer intervention or referral to appropriate therapy is key to optimizing overall patient health outcomes. In fact, all nurses interviewed emphasized the connection between mind and body and how they affect each other. In addition, nurses expected that standardized screening would help normalize mental disorders and encourage patients to seek professional help.

Regarding the nurse–patient relationship, it is commonly known that nurses have the most contact time to inpatients (36), so it is not surprising that most nurses interviewed considered themselves to be the right point of contact to deliver screening. Nurses described that screening questions serve as icebreakers and that sharing such intimate feelings intensifies the relationship with the patient. According to literature, the nurse–patient relationship is key to treatment success (37), as the patient–nurse relationship, and the quality of care, interrelate (38). This special relationship empowers nurses to significantly influence patients’ affective complaints (39).

Although the workload of nurses has predominantly increased (40), most of the nurses interviewed stated that the screening process was a relief for them. First, collaboration with professional colleagues was perceived as facilitating. Results that were also found in a study with a similar collaborative team approach in primary care (41). Second, the enhancement of self-efficacy through nurse participation was found to be beneficial. Indeed, a significant positive relationship was found between nurses’ professional self-concept regarding what they do and why they do it and their job satisfaction (42). However, our resulats indicate that questions about psychological burden can be perceived as unpleasant. Both from the perspective of the questioner and the respondent. We suspect that nurses feel uncomfortable because they may not feel adequately trained to ask questions about mental health and to deal with patients’ potentially emotional responses. For example, only half of nurses reported having been trained in the use of the PHQ-4, although few nurses reported feeling inadequately trained. In this context, other studies emphasize the importance of good implementation of complex medical interventions (43, 44). In this case, implementation occurred as early as 2017, so ongoing training might be useful in addition to good implementation like stated by Norouzinia, Aghabarari (45). This training could, for example, compensate for staff turnover and a possible loss of knowledge in the care team.

According to our results, nurses suspect two different reasons why primarily somatic inpatients decline further psychosomatic treatment. Either no further referral is needed because the patient has other resources, such as family, or no further treatment is desired. This issue is more common in cancer research. For example, Carlson (46) emphasizes the emotional component of why someone does not want treatment and, consistent with our findings, advocates the approach of clarifying misunderstandings, e.g., due to lack of information, and consequently addressing the reasons for refusal. However, we could not find concrete reasons for refusal of psychosomatic treatment in primarily somatic, non-oncological patients in literature.

The interviewed nurses indicated that application requirements should be modified to facilitate delivery. As with Colligan et al. (47), barriers to patient communication were a recurring challenge. Some nurses interviewed felt that the structure of the screening questions was not optimal to be asked orally or that the questions themselves should be more patient-friendly, as patients sometimes struggled understanding content and language.

## Limitations

First, data were collected in only one hospital, which limits the external validity of this study. Second, the recruitment method may affected the sample selection because we relied on voluntary participation and represents only nurses that were willing to share their experiences. Third, we interviewed more women than men; although this seems to reflect the actual gender distribution in the nursing profession. Fourth, due to the anonymization process, some valuable personal information about the nurses interviewed (e.g., gender, ward, or employment status) could not be provided. Fifth, we did not interview other medical personnel, such as the consultant service, or the patients who underwent the intervention. Thus, we were able to gain only a limited perspective on this complex intervention.

## Conclusion

None of the nurses interviewed opposed standardized nurse-led screening for mental comorbidities and related psychosomatic consultation on somatic wards. Every nurse interviewed would like to continue using it in the future and indicate its meaningfulness on a variety of levels. The participating nurses promoted the holistic approach and should be empowered in their own skills and competencies, leading to greater job satisfaction and self-efficacy among nurses. However, to take full advantage of these opportunities, usability improvements, regular supervision, and ongoing training should be considered. Ultimately, the nurses’ experiences is invaluable to further improve interdisciplinary care.

### Relevance to clinical practice (60 words)

Nurses endorse standardized nurse-led screening for mental comorbidities and related psychosomatic consultation on somatic wards. The intervention has the potential to provide a holistic approach to patient care while strengthening nurses’ skills and competencies, leading to greater job satisfaction and self-efficacy. To take full advantage of this potential, usability adjustments, regular supervision, and ongoing training for nurses should be considered.

## Data availability statement

The datasets presented in this article are not readily available because of ethical and privacy restrictions. Requests to access the datasets should be directed to the corresponding author.

## Ethics statement

The studies involving human participants were reviewed and approved by Approved by the ethics committee of the University Medical Center Hamburg-Eppendorf, Germany (reference number: LPEK-0181). The patients/participants provided their written informed consent to participate in this study.

## Author contributions

L-EB and SK had full access to all data in the study and take responsibility for the integrity of the data and the accuracy of the data analysis. L-EB, SK, SL, JS, and BL: study concept and design. JS and L-EB: acquisition of data. L-EB, JS, and SK: analysis and interpretation of data. L-EB and SK: drafting of the manuscript. SL and BL: critical revision of the manuscript for important intellectual content. L-EB, SK, SL, JS, and BL: administrative, technical, or material support. SK: study supervision. All authors contributed to the article and approved the submitted version.

## Funding

We acknowledge financial support from the Open Access Publication Fund of UKE - Universitätsklinikum Hamburg-Eppendorf and DFG – German Research Foundation.

## Conflict of interest

The authors declare that the research was conducted in the absence of any commercial or financial relationships that could be construed as a potential conflict of interest.

## Publisher’s note

All claims expressed in this article are solely those of the authors and do not necessarily represent those of their affiliated organizations, or those of the publisher, the editors and the reviewers. Any product that may be evaluated in this article, or claim that may be made by its manufacturer, is not guaranteed or endorsed by the publisher.
