# Supplementary material for: Nurses’ experiences of a screening and associated psychosomatic consultation service for mental comorbidities in somatic care inpatients – a qualitative study
Source: Front Psychiatry. 2023 Jun 2;14:1148142. doi: 10.3389/fpsyt.2023.1148142 (PMC10272840; doi:10.3389/fpsyt.2023.1148142)
Supplement: Supplementary file 1 [file Table_1.docx]

**Consolidated criteria for reporting qualitative studies (COREQ): 32-item checklist**

For *Nurses´ perspective of a screening and associated psychosomatic consultation service for mental comorbidities in somatic care inpatients – a qualitative study.*

| **No** | **Item** | **Guide questions/description** | **Details for our study** |  |  |  |
| --- | --- | --- | --- | --- | --- | --- |
| **Domain 1: Research team and reflexivity** | | |  |  | |  |
| Personal Characteristics | | | |  |  |  |
| 1. | Interviewer/facilitator | Which author/s conducted the interview or focus group? | LEB and JS |  |  |  |
| 2. | Credentials | What were the researcher's credentials? E.g. PhD, MD | LEB: MSc. Psychology and PhD cand.; JS: MD cand. |  |  |  |
| 3. | Occupation | What was their occupation at the time of the study? | LEB: Researcher; JS: medical student |  |  |  |
| 4. | Gender | Was the researcher male or female? | LEB: female; JS: male |  |  |  |
| 5. | Experience and training | What experience or training did the researcher have? | LEB: MSc. Psychology, experienced with qualitative research and interview techniques; JS: trained in interview techniques |  |  |  |
| Relationship with participants | | | |  |  |  |
| 6. | Relationship established | Was a relationship established prior to study commencement? | No. |  |  |  |
| 7. | Participant knowledge of the interviewer | What did the participants know about the researcher? e.g. personal goals, reasons for doing the research | Nurses were informed that the study contributes to the scientific work of LEB (PhD cand.) and JS (MD cand.). |  |  |  |
| 8. | Interviewer characteristics | What characteristics were reported about the interviewer/facilitator? e.g. Bias, assumptions, reasons and interests in the research topic | The nurses were informed that the study provides feedback to the interdisciplinary working group of the screening service. |  |  |  |
| **Domain 2: study design** | | | |  |  |  |
| Theoretical framework | | | | | |  |
| 9. | Methodological orientation and Theory | What methodological orientation was stated to underpin the study? e.g. grounded theory, discourse analysis, ethnography, phenomenology, content analysis | Thematic analysis by Braun and Clarke (2006), within the framework of an essentialist approach. |  |  |  |
| Participant selection | | | |  |  |  |
| 10. | Sampling | How were participants selected? e.g. purposive, convenience, consecutive, snowball | Every nurse working on the cooperating internal medical and dermatological wards was invited to participate. |  |  |  |
| 11. | Method of approach | How were participants approached? e.g. face-to-face, telephone, mail, email | We promoted the project during team meetings and direct inquiries. |  |  |  |
| 12. | Sample size | How many participants were in the study? | 19 nurses. |  |  |  |
| 13. | Non-participation | How many people refused to participate or dropped out? Reasons? | One nurse refused after written consent due to doubts about the anonymizazion process. |  |  |  |
| Setting | | | |  |  |  |
| 14. | Setting of data collection | Where was the data collected? e.g. home, clinic, workplace | In the clinic (University Medical Center Hamburg-Eppendorf, Germany). |  |  |  |
| 15. | Presence of non-participants | Was anyone else present besides the participants and researchers? | No. |  |  |  |
| 16. | Description of sample | What are the important characteristics of the sample? e.g. demographic data, date | Nurses working on the cooperating internal medical and dermatological wards conducting the nurse-led screening. |  |  |  |
| Data collection | | | |  |  |  |
| 17. | Interview guide | Were questions, prompts, guides provided by the authors? Was it pilot tested? | We developed a semi-structured interview guide. It was not pilot tested. |  |  |  |
| 18. | Repeat interviews | Were repeat interviews carried out? If yes, how many? | No. |  |  |  |
| 19. | Audio/visual recording | Did the research use audio or visual recording to collect the data? | Audio records. |  |  |  |
| 20. | Field notes | Were field notes made during and/or after the interview or focus group? | No. |  |  |  |
| 21. | Duration | What was the duration of the interviews or focus group? | The interviews took 30 – 40 minutes, each. |  |  |  |
| 22. | Data saturation | Was data saturation discussed? | In contrast to grounded theory, data saturation is not a construct targeted for thematic analysis and therefore was not discussed. |  |  |  |
| 23. | Transcripts returned | Were transcripts returned to participants for comment and/or correction? | No. |  |  |  |
| **Domain 3: analysis and findings** | | | | | |  |
| Data analysis | | | |  |  |  |
| 24. | Number of data coders | How many data coders coded the data? | LEB coded the data. Consensus was reached in discussion with JS and SK. |  |  |  |
| 25. | Description of the coding tree | Did authors provide a description of the coding tree? | No, due to language difference. The study was conduced in German. |  |  |  |
| 26. | Derivation of themes | Were themes identified in advance or derived from the data? | Themes derived from the data. |  |  |  |
| 27. | Software | What software, if applicable, was used to manage the data? | MAXQDA software (version 2020). |  |  |  |
| 28. | Participant checking | Did participants provide feedback on the findings? | No. |  |  |  |
| Reporting | | | |  |  |  |
| 29. | Quotations presented | Were participant quotations presented to illustrate the themes / findings? Was each quotation identified? e.g. participant number | Yes, each quote is cited with an annonymized participant number. |  |  |  |
| 30. | Data and findings consistent | Was there consistency between the data presented and the findings? | Yes, findings were reported in consistency with the data. |  |  |  |
| 31. | Clarity of major themes | Were major themes clearly presented in the findings? | Yes. We developed eight themes in consistency with our data and enriched every theme and its codes with quotations. |  |  |  |
| 32. | Clarity of minor themes | Is there a description of diverse cases or discussion of minor themes? | Yes. For example the theme “psychological effects “ |  |  |  |

The checklist is provided by Tong, Sainsbury and Craig (2007).

Published: Tong, A., Sainsbury, P., & Craig, J. (2007). Consolidated criteria for reporting qualitative research (COREQ): a 32-item checklist for interviews and focus groups. International journal for quality in health care, 19(6), 349-357.
